# An Unusual Cause of Ascites in Liver Cirrhosis: Peritoneal Tuberculosis

**DOI:** 10.7759/cureus.12505

**Published:** 2021-01-05

**Authors:** Rupinder Mann, Abhishek Gulati

**Affiliations:** 1 Internal Medicine, Saint Agnes Medical Center, Fresno, USA; 2 Gastroenterology, Saint Agnes Medical Center, Fresno, USA

**Keywords:** : tuberculosis, serum ascites albumin gradient, alcohol-related cirrhosis, bacterial peritonitis

## Abstract

Peritoneal tuberculosis is a rare disease with increasing incidence in recent years, especially in patients with an immunocompromised state and liver cirrhosis. We report the case of a 37-year-old male with a known history of liver cirrhosis who presented to the hospital with abdominal pain, abdominal distension, and was diagnosed with peritoneal tuberculosis. The diagnosis was made based on findings from a CT of the abdomen and histopathological findings of peritoneal tissue biopsy. He was started on ethambutol, isoniazid, pyrazinamide, and rifampin for six months.

## Introduction

Extrapulmonary tuberculosis accounts for 18.7% of all tuberculosis (TB) cases in the United States (US), and peritoneal tuberculosis constitutes only 4.7% of all cases of extrapulmonary tuberculosis [1]. Patients with liver cirrhosis are at an increased risk of developing tuberculous peritonitis (TBP). TBP in cirrhotic patients can mimic spontaneous bacterial peritonitis (SBP) and is infrequently considered in the differential diagnosis, resulting in delayed diagnosis and consequent mortality [2]. In this report, we present a case of TBP in a patient with liver cirrhosis.

## Case presentation

**Figure 1:**
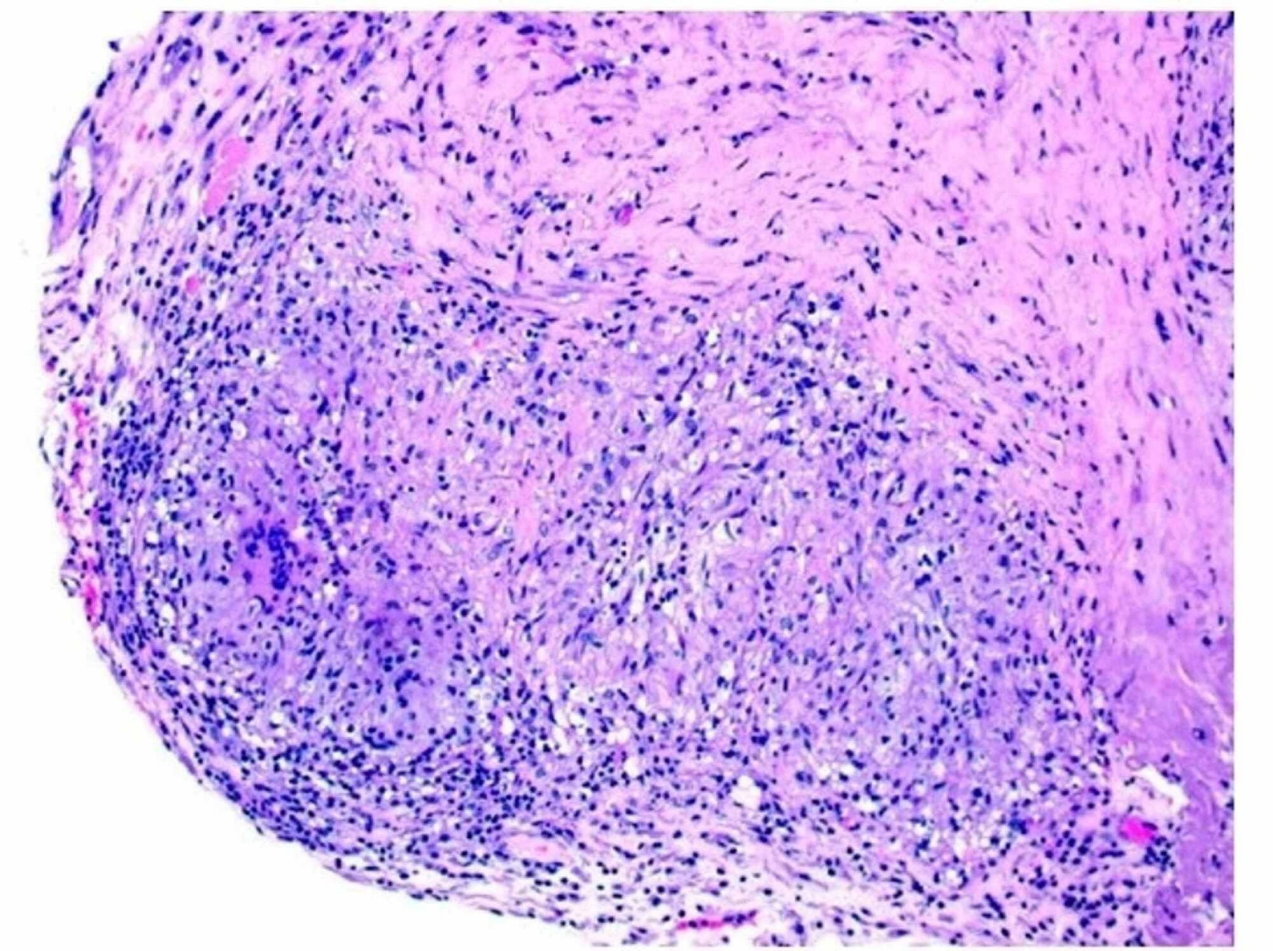
Peritoneum biopsy (10X) showing granulomatous inflammation with caseous necrosis

**Figure 2:**
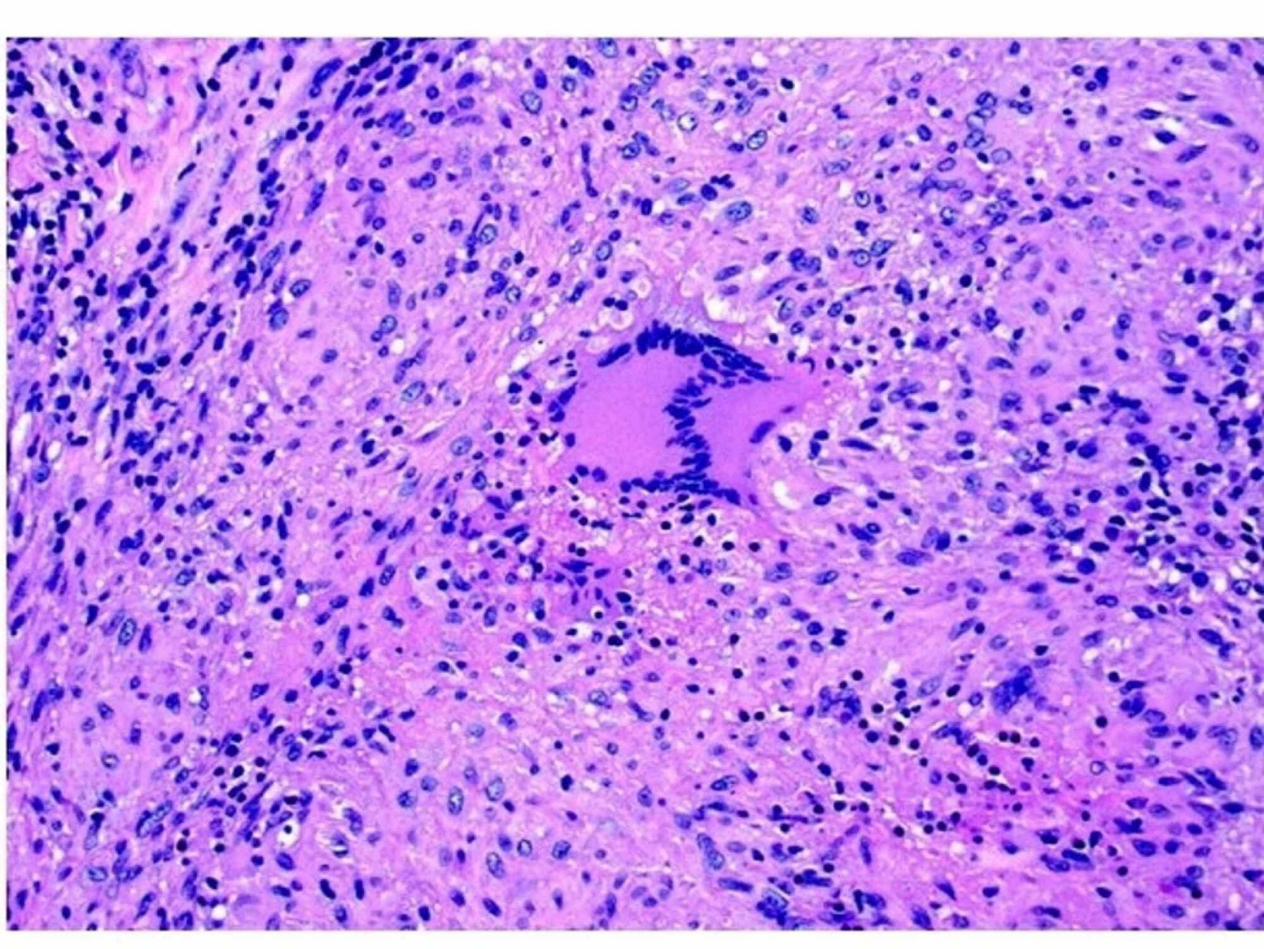
Peritoneal fat tissue excisional biopsy (20X) showing granulomatous inflammation with caseous necrosis

A 37-year-old man with a past medical history of alcoholic liver cirrhosis and ongoing alcohol abuse presented to the emergency department with decreased appetite, gradually worsening abdominal distension for three to six months, and epigastric abdominal pain for two days. The patient also had a history of pulmonary tuberculosis over 20 years ago and had been treated with a multi-drug regimen for six months. On admission, the patient’s vital signs were stable, and on the exam, he was noted to have epigastric tenderness and abdominal distension. Lab data were pertinent for sodium (Na) of 124 mmol/L (normal range: 135-145 mmol/l), aspartate transferase (AST) of 61 U/L (normal range: 6-35 U/L). His white blood cell (WBC) count was 7.9. CT of the abdomen demonstrated moderate ascites with thin peripheral enhancement suggestive of peritonitis. The patient was started on empiric antibiotics for presumed SBP. Paracentesis removed 1.4 liters (L) of straw-colored fluid with WBC of 791 cells/L with a lymphocytic predominance (94%), with low serum-ascites albumin gradient (SAAG) of 0.4 g/dL. Gram stain and ascitic fluid culture came back negative. Ascitic fluid cytology showed benign mesothelial cells and mildly increased small mature lymphocytes. The patient continued to have a low-grade fever of up to 100.4 °F during the hospital stay. Given the continued low-grade fevers and lymphocytic predominance in the ascitic fluid, serum QuantiFERON gold was ordered, which came back positive. A laparoscopic peritoneal biopsy was recommended after consultation with an infectious disease specialist. Laparoscopy demonstrated exudates and loculated ascites. A peritoneal biopsy was performed, which showed granulomatous inflammation with caseous necrosis and confirmed *Mycobacterium tuberculosis* complex infection (Figure 1, Figure 2). The patient was started on the four-drug regimen for TB treatment with ethambutol, isoniazid, pyrazinamide, and rifampin for six months.

## Discussion

TBP should be considered as a differential diagnosis in addition to SBP in cirrhotic patients who present with ascites and abdominal pain. Similar to SBP, TBP in patients with cirrhosis presents with nonspecific signs and symptoms including abdominal distension, fever, abdominal pain, and diarrhea [2]. However, treatment of TBP and SBP differs vastly from each other. In most cases, immunocompromising states predispose patients to peritoneal tuberculosis, and the risk factors include liver cirrhosis, diabetes mellitus, use of systemic corticosteroids, HIV infection, and underlying malignancies [3]. The clinical presentation can be acute or chronic. Abdominal pain is the most common presenting symptom (80-95%) followed by fever (40-90%), weight loss (40-90%), diarrhea (11-20%), anorexia, and malaise [3]. Adhesions may also cause small intestinal obstruction. On physical examination, diffuse abdominal tenderness, doughy abdomen, hepatomegaly, and ascites may be present [4]. Diagnostic testing includes paracentesis with acid-fast bacilli (AFB) stain and culture, imaging (CT or ultrasound), and peritoneal biopsy. Paracentesis most often demonstrates exudative, lymphocytic fluid. A diagnostic gold standard is the culture of *Mycobacterium* from ascitic fluid or peritoneal biopsy. However, ascites culture and smear have low sensitivity (<20%). Laparoscopy can reveal the typical peritoneal studding of the tubercles and yield tissue for culture and smear. Visualization of tubercles during laparoscopy can be diagnostic in up to 95% of cases, and biopsies are nearly 100% sensitive in obtaining a diagnosis [3]. Although ascitic adenosine deaminase (ADA) has been used as a rapid test for the diagnosis of TBP, its role in the setting of cirrhosis is controversial [2]. In developed countries with a low incidence of TB and a high prevalence of cirrhosis, ascitic fluid ADA has been found to be good in terms of accuracy but has shown low sensitivity and imperfect specificity [1,2]. However, these observations were countered by Liao et al., who reported that ADA activity showed high sensitivity and specificity in a cirrhotic patient when using a cut-off value of >27 U/l [2]. TBP should be considered with the following criteria: cirrhotic patients with Child-Pugh class B; TB identified at additional sites; lymphopenia in the peripheral blood; an ascitic protein concentration of >25 g/l; a predominance of lymphocytes in ascites; ascitic ADA activity levels of >27 U/l; and ascitic lactic dehydrogenase (LDH) levels of >90 U/l [1].

Treatment of TBP is pharmacological with anti-TB medications for at least six months including an initial two months of four-drug regimen followed by a two-drug regimen [4]. Of note, 20-40% of patients with abdominal TB present with an acute abdomen and need surgical intervention. Surgical intervention is also reserved for patients who develop complications of abdominal TB including ulcer, perforation with abscess or fistula, massive bleeding, complete obstruction, or obstruction not responding to medical management. TBP generally responds to medical treatment, and early diagnosis and management can prevent unnecessary surgical interventions [1,4].

This article was previously presented as a meeting abstract at the 2019 American College of Gastroenterology (ACG) Annual Scientific Meeting on October 29, 2019, in San Antonio, TX.

## Conclusions

This case report highlights how the diagnosis of TBP can be challenging. TBP should be part of the differentials for alcoholic liver disease patients presenting with ascites since a delay in diagnosis can impede the treatment. TBP and SBP have different treatment methods, and without treatment, TBP can lead to significant morbidity and mortality.
